# The Value of Intravenous Immunoglobulin Therapy in Idiopathic Inflammatory Myositis in the Current Transformed Era of Biologics

**DOI:** 10.7759/cureus.7049

**Published:** 2020-02-19

**Authors:** Anjali Patwardhan

**Affiliations:** 1 Child Health, University of Missouri, Columbia, USA

**Keywords:** biologics, juvenile dermatomyositis, interstitial lung disease, rituximab, dysphagia, idiopathic inflammatory myopathy, polymyositis, recalcitrant

## Abstract

The understanding of etiology and pathogenesis of idiopathic immune myositis is fast evolving, and so is the classification of myositis subtypes. The diversity in genetics, major histocompatibility complex expressions, immunohistochemical, and specific and associated autoantibodies not only explains the individual variability in response to therapies but also begs for subtype-specific treatments. With the evolution of the new biological therapies, the treatment of idiopathic immune myositis (IIM) has greatly transformed in recent years. This article appraises the current therapeutic value of intravenous immunoglobulin (IVIg) in idiopathic immune myopathy patients in the era of transformed treatment options. This article argues why the IVIg therapy still retains its value as an unreplaceable treatment option in certain specific subtypes of idiopathic immune myositis patients as well as in certain specific clinical idiopathic immune myositis scenarios.

## Introduction and background

Idiopathic immune myositis (IIM) syndromes are considered complement-mediated microangiopathies that involve small vessels of muscles, skin, and internal organs and cause ischemic damages. Several pieces of evidence, such as the presence of myositis-specific and associated autoantibodies (MAS, MSA), infiltration of tissues with immune cells, and the overexpression of major histocompatibility complex (MHC, class I and II) on myofibrils, point to the autoimmune origin of IIM [1]. Recent evidence also points to the inappropriate stimulation of the innate immune system (interferons and IFN-regulated proteins), leading to the dysregulation of the adaptive immune retort through dendritic cells. The interferons and IFN-regulated proteins are believed to have etiopathologic role especially in dermatomyositis (DM) and juvenile dermatomyositis (JDM) [2]. Roifman et al. first time reported a drastic improvement in a JDM patient with intravenous immunoglobulin (IVIg) who had failed steroids, methotrexate, and cyclophosphamide therapy [3]. The exact mechanism of action of IVIg is still not clear and considered multifactorial. There are several theories on how the IVIg works in myositis patients, such as it acts as immunomodulatory drug/an immune booster, reduces the production of autoantibodies, works through complement fixation, neutralizes the assailant autoantibodies, or autoantigens, causes cytokines suppression or blockage [4]. IVIg is known to inhibit IL-2, IL-10, TNF-𝛽, and IFN-𝛾, derived from T-cells. The IVIg may effect through its suppressing actions on the dendritic cells and their maturation [4]. It blocks the Fc-receptors on autoantibodies, therefore, prevents the phagocytosis of antibody-coated cells [5].

The anti-inflammatory effect of IVIg on autoantibody-induced inflammation may be due to its ability to induce expression of the inhibitory Fc and Fc- y- RIIB receptors. Apart from all the previously mentioned actions, IVIg also has an immediate and long-lasting attenuating effect on complement amplification by stimulating inactivation of C3 convertase precursors [5].

The utility of IVIg therapy is especially found unparalleled in situations where immunosuppressants are contraindicated, such as pregnancy and fulminant infections. IVIg has been selectively used in some specific clinical situations and has shown comparative therapeutic superiority over other therapeutic agents such as myositis with lung and esophageal involvement, as detailed later in this article. The safety profile and low adverse effects of IVIg, as compared to other immunosuppressants and biologics, made it popular drug in the treatment of IIM, despite the rising costs, supply shortages and still not being FDA-approved therapy for myositis. Most immunosuppressants except the pulse steroids need a latent period before the clinical effect can be seen. The IVIg and cyclophosphamide are the drugs that need a variable but relatively short, i.e., in weeks rather than in months, latent period for clinical results. This article explores the utility and current value of IVIg in patients with myositis.

## Review

Efficacy and use of IVIg in IIM

Other than steroids, high dose IVIg is the only drug that is researched and found to be effective for the treatment of IIM in a double-blind and placebo-controlled trial [6]. Several studies report the successful use of IVIg in different subgroups of IIM in diverse clinical settings as listed in the following section.

Two randomized controlled trials (RCT) and several prospective uncontrolled studies have reported the successful use of high dose IVIg in DM and polymyositis (PM) patients who had failed the therapy with steroids and at least one disease-modifying anti-rheumatic drug (DMARD) [7, 8]. The response and efficacy of high dose IVIg in inclusion body myositis (IBM) patients are not well established [9]. In the controlled cross-over design double-blind, placebo-controlled study, the sporadic inclusion body myositis (s-IBM) patients showed only marginal clinical improvement with high dose IVIg [10]. One case report of successfully treating IBM patients with the low dose IVIg begs further exploration in low dose therapy [11]. Binns et al. report a good response to IVIg with rituximab and cyclophosphamide in a three anti-signal recognition particle-associated JDM (anti-SRP JDM) patients [12]. The positive response to rituximab in anti-SRP JDM patients is already recognized. Therefore, the contribution of IVIg in the combined success when used with rituximab is difficult to assess [13]. It is worth mentioning that anti-SRP JDM, along with anti-HMG-CoA-reductase (anti-HMGCR) JDM, is a distinct and severe form of necrotizing muscle predominant and resistant to treat JDM [14]. The IVIg is particularly favored and has shown significant success in skin predominant and or resistant to treat skin diseases in JDM patients [15]. Several researchers have reported significant response to high dose IVIg in their refractory/steroid-resistant/steroid-dependent classic JDM patients [16]. Imataka and Arisaka reported successful treatment of a steroid-refractory Banker-type JDM in two-year-old patients with IVIg (400 mg/kg/five days a week/six week for six cycles) [17]. In the largest cohort that received IVIg, Lam et al. reported that IVIg showed better results in steroid-resistant patients than in steroid-dependent patients, but steroid-dependent patients showed lower disease activity than the control JDM patients who did not receive IVIg. They also reported the best response in SR JDM patients [18]. Al-Mayouf et al. reported a steroid-sparing effect in their 18-steroid dependent JDM patients who received treatment with IVIg [16]. In modern medicine, the use of IVIg is well recognized and prevalent as a second/third line conventional therapy for IIM.

The experience is wide-ranging when IVIg is used as first-line therapy. Some researchers believe that IVIg should be used as first-line therapy in DM/PM-ILD (interstitial lung disease) patients, while others used effectively in refractory patients [19-21]. The higher medication cost, as well as risk for exposure to blood-borne diseases, is the prohibiting factors for the use of IVIg as the first-line therapy. The cumulative cost vs. comparative quality analysis (repeat therapies, hospital stay, work/school days lost, quality of life, the dollar value of the therapy) of various therapeutic options for myositis is not performed. The following are some of the experiences where IVIg was used as first-line therapy. IVIg as first-line therapy has been reported to be unsuccessful in an open-label study of 11 patients (5/11 PM and 6/11 DM) [8]. Diot et al. and few other researchers recommend IVIg as first-line therapy in myositis patients but along with other immunosuppressants, more so if the patient has interstitial lung disease (ILD) and or dysphagia also [19-21]. Some researchers recommend IVIg as first-line therapy in patients with esophageal involvement [20].

There are some studies where IVIg is used in ILD-associated PM/DM. ILD is considered as a poor prognostic factor and predictor of high mortality despite aggressive therapy in patients with myositis [21]. It is recognized now that the interstitial inflammation may be part of the initial disease process, which may go undetected if not careful. Certain factors such as anti-MDA5 antibody positivity and hyperferritinemia are not only the risk factors for developing ILD but also predict its expansion to rapidly progressive ILD [22]. The manifestations, diseases course, pathophysiology, immunologic profiles, histopathology findings and outcomes of ILD have been heterogeneous and different in subgroups of IIM suggesting variations in immunopathogenic mechanisms [23]. High/persistent fever at presentation (known as Hamman-Rich-like presentation), hypoalbuminemia, cardiac involvement, amyopathic diseases with interstitial pneumonia, are bad prognostic factors for development and progression of PM/DM-associated ILD [24]. The presence of ILD which also correlates with the severity of Gottron's papules/rash, cardiac involvement, the age of onset at 40 years or above, arthralgia, pulmonary fibrosis, and fevers are considered poor diseases prognostic factors in all the subgroups of IIM (JDM/DM/PM) [25].

Some serologic markers such as serum KL-6 level, serum surfactant protein-D (SP-D), serum surfactant protein-A (SP-A), combined SP-D and SP-A, may be potentially useful serum markers for the diagnosis of ILD in PM/DM patients.

The serum SP-A is a prognostic factor, while serial serum KL-6 levels are also used to monitor response to the therapy in PM/DM-ILD in patients [26]. Suzuki et al. described their retrospective experience (1985-2007) with five PM/DM/amyopathic dermatomyositis (AMD) patients (one PM, four ADM with one positive for anti-Jo-antibody, three females, two males) with severe resistant to treat ILD with similar clinical courses, parameters, and demography. These five patients were treated with IVIg after they had failed conventional therapy, including cyclosporine and cyclophosphamide. They had a total of 57 patients admitted with PM/DM with ILD during the said period, but only five who did not respond to high dose steroids and one more immunosuppressant were labeled as ‘resistant’ and were treated with IVIg as ‘salvage therapy.’ Only 2/5 patients (one PM, one ADM) survived [27]. Fujisawa et al., in their series of 28 patients (16 PM-ILD, 12 DM-ILD) identified that ILD-DM patients are more likely to be corticosteroid-refractory than ILD-PM and have a worse prognosis [28]. Ye et al., in their series of 145 patients (1998-2005), not only confirmed findings of Fujisawa et al. but also noted that the worst outcome was in AMD-ILD followed by DM-ILD and the best outcome amongst the three subgroups was in PM-ILD [29]. The anti-melanoma differentiation-associated gene five antibodies (anti-MDA5) positive patients are reported to have a rapidly progressing disease course followed by death [22]. The anti-aminoacyl-tRNA synthetase (anti-ARS) positive patients respond to corticosteroids early in the diseases, but then frequently relapse and run a progressive course and finally may become refractory to steroid therapy over time [22]. The anti-ARS antibody-positive patients comparatively do better than anti-MDA5 positive patients [22]. The difference in various ratios of IL-4, IFN-γ, and serum IL-8 levels is believed to be the cause of pathophysiologic differences in anti-ARS-ILD and anti-MDA5-ILD [30].

The early recognition and appropriate selection of the correct subtype of the patient are vital to select appropriate therapeutic strategies.

Apart from IVIg, the cyclosporine and tacrolimus had been used selectively successfully in ILD+PM/DM patients [31].

IVIg in dysphagia associated with myositis

As a part of systemic myositis, the cricopharyngeal, oropharyngeal, sternomastoid muscle, and esophageal muscles may also get involved leading to difficulty in swallowing, achalasia, aspiration, voice change and associated complications [32]. The dysphagia occurs in a wide range of IIM patients (10-73%) and can contribute to a poor outcome and high mortality [33]. The new development of dysphagia can either suggest the progression of disease relapse.

Mugii et al., in their 92 adult Japanese DM patients, reported that the positive anti-TIF-1γ antibody, malignancy-comorbidity, higher age, male sex, lower mean MMT scores of sternomastoid muscle, and interstitial pneumonia are strongly associated with the likelihood of developing dysphagia in adult myositis patients. These all risk factors are independently also considered as bad prognostic features [34].

The prevalence of dysphagia in JDM is not precisely reported as extensively as in adults. There are technological barriers in the interpretation of the videofluoroscopic swallowing study (VFSS) in children. Despite the paucity of data, most researchers agree that dysphagia in childhood PM/JDM is a worse prognostic marker and associated with a higher mortality rate [35]. As opposed to adults, researchers have yet not identified any correlation between VFSS scores and other disease activity indices (MMT/CMAS, physician VAS, CHAQ) in pediatric myositis patients. Another difference in pediatric myositis was that the clinical and laboratory markers could not predict the abnormal swallowing scores by VFSS, suggesting that dysphagia and rest of the JDM disease activity may not run hand in hand [36].

Taieb et al., in their 70 JDM patient series, concluded that dysphagia was independently a worse prognostic factor, and patients with dysphagia had higher mortality rates than those without [37]. In the UK and Ireland National Registry/Repository for JDM, 33/114 (29%) patients had symptomatic dysphagia, and 22/126 (17%) had symptomatic dysphonia [38]. Chiu et al. reported dysphagia in 19% of their series of 32 JDM patients [39]. In McCann’s series of 14 JDM patients, two asymptomatic patients had abnormal, and three symptomatic patients had normal VFSS. De Inocencio et al. reported reduced side effects and better tolerability and effectiveness of subcutaneous (SC) immune globulins with high-dose recombinant human hyaluronidase. The experience comes from their five juvenile dermatomyositis patients who could not tolerate high dose IVIg due to side effects such as nausea, vomiting, severe headaches, or who had poor venous access. They have reported comparable serum IgG levels after SC IVIg with dose adjustments (fSCIg 1 g/kg on days 1 and 6 of every 28-day cycle) [40].

Marie at al., in the retrospective chart review of 73 adult refractory myositis patients (39/73 PM, 34/73 DM) with dysphagia, reported their experience with high dose (2 g/kg/dose/month) IVIg therapy. They not only reported good outcomes but also recommend the combination of IVIg and high-dose steroids as the first-line therapy in life-threatening cases of dysphagia [19]. Earlier, Marie et al. had reported a 100% success and remission in their three severely dysphagic steroid-resistant DM/PM patients with three doses of IVIg [41]. Several reports confirm the sustained response to high dose IVIg in patients with PM/DM dysphagia [41]. In these reports, the IVIg was used successfully with or without other DMARDs, methylprednisolone and oral steroids in DM/PM patients who were poor steroid responders, steroid-refractory or dependent and had rapidly progressive life-threatening dysphagia.

Unlike other IIM, dysphagia in IBM is more common and resistant to steroid therapy, which makes it difficult to treat [42]. Experience with IVIg is varied amongst different researchers. Jones et al. performed a detailed Cochrane review and concluded that there is no significant value of using IVIg in IBM patients with dysphagia [43]. In a double-blind, placebo-controlled crossover study in 19 IBM patients with dysphagia (age 42-74 years) with average disease duration of 5.6 years (range 3-10 years), the patients received monthly infusions of 2 g/kg IVIg/placebo for three months. Though the study failed to show a muscle strength improvement in the treatment arm, a mild improvement in swallowing duration (in seconds) measured with ultrasound was reported in the treatment arm (p < 0.05) [3]. Cherin et al. showed sustained improvement in dysphagia with subcutaneous Ig (SCIg) in their six patients with IBM, though relapses were frequent. The longest duration of sustained improvement in their series was 12 months [44].

Summary of IVIg therapy in IIM

Except for IBM, IVIg therapy is considered a safe and effective therapy for most subgroups of IIM patients. It is known to produce relatively early and sustained improvement, especially in DM/JDM patients. IVIg is especially useful with patients with ILD, lung involvement, skin-predominant disease, in the setting of infection, and in patients with swallowing difficulties. The usual dose is 2 g/kg/month, but it has been used variably by different researchers. Promising results are reported even after low dose as well as high dose subcutaneous IVIg when given intermittently or through a programmable pump [44]. Significant improvements in muscle strengths, muscle enzyme levels, quality of life and steroid-sparing effect have been reported in most DM/JDM patients who were considered refractory. The guidelines of the American Academy of Neurology (2012) suggest that there is no conclusive evidence to support the use of IVIg in polymyositis patients [45]. It is an expensive drug that prohibits its use as a first- or second-line therapy or even as a long-term therapy. Currently, the IVIg commonly is utilized as salvage therapy in refractory patients, but its early use in the specific clinical scenarios such as myositis with ILD, myositis with dysphagia, myositis with sepsis, and skin predominant refractory disease should be encouraged.

Some researchers recommend IVIg therapy as first-line therapy in viral or drug-induced myopathy which closely resembles inflammatory myopathy, such as due to mycoplasma and several viruses-induced myopathies [8].

IVIg in skin predominant IIM

The skin component of DM or JDM could be more recalcitrant to treatment than that of muscle disease and can have a severe disability, morbidity, and quality of life (QoL) issues [46]. The skin disease could be in pre-muscle disease, post- muscle disease, predominant skin disease, or skin only dermatomyositis/JDM (amyotrophic myositis). Aggressive sun protection (more than 70% SPF) is universally advised along with other therapies. The topical approaches such as antipruritic agents, tacrolimus ointment, steroid ointments and oral antimalarial (hydroxychloroquine 5 mg/kg/day, maximum 400 mg, quinacrine, and chloroquine) are added to the systemic therapy as add-on or sole therapies based on the severity of diseases as measured by the Cutaneous Dermatomyositis Disease Area and Severity Index (CDASI: 0 to 100). The second line treatment includes DMARDs such as methotrexate (MTX) at the dose of 15 mg/m^2^ or 1 mg/kg once/week, maximum 40 mg, mycophenolate mofetil (MMF) at the dose of 600 mg/m^2^, or azathioprine (AZA) at the dose of 1 to 2 mg/kg/day. The cyclosporine is used as a bridge short-term treatment. The intravenous immunoglobulin (IVIg) therapy (2 g/kg/day spread over 2-5 days) is reserved for the refractory skin diseases myositis patients. Therapies such as dapsone and thalidomide are considered in exceptional circumstances weighing the risk vs. benefits. The rituximab has been used if the patient has failed all the above-listed medications. Anecdotal cases of treatment of recalcitrant cutaneous diseases in myositis by total body irradiation, stem cell transplantation, and plasmapheresis have been reported [47].

IVIG reduces the concentration of MAC deposits in the skin as well as myofibril capillaries, downregulates T cells, reduces the adhesion molecules expression (ICAM-I, VCAM, TGF-βand MHC-I molecules) and down-regulates cytokine production, in myositis patients [48]. The IVIG treatment is effective both for the skin as well as muscle disease in DM, JDM, and some polymyositis patients.

Value of IVIg in myositis calcinosis cutis and calcinosis

Abnormal deposition of calcium salts in skin, subcutaneous tissue (calcinosis cutis), and muscles is called calcinosis, which can be dystrophic, idiopathic, iatrogenic, transplant-related, tumoral calcinosis or metastatic. The calcinosis in the presence of normal serum calcium and phosphate levels is called dystrophic calcinosis.

The jury is still out for the answers if calcinosis is a part/signature of the myositis diseases itself or occurs as a result of the nonspecific chronic micro-injury and inflammation. There is no structured trial, but several reports support the value of IVIg in IIM patients with calcinosis.

Shahani reports successfully treating a 30-year-old patient with DM with painful calcinosis who failed steroid, and several DMARDs and responded to 2 gm/kg/month-IVIg over two consecutive days [49]. A survey on pediatric rheumatologist suggested that just over 1/4th rheumatologists perform initial screening imaging for calcinosis in newly diagnosed IIM patients. Sixty-seven percent of survey responders preferred to step up the immunosuppression as the first step for calcinosis, 13% did not do something specific, but continue with the ongoing therapy for IIM, 13% add other ‘non-immunosuppressive’ therapies. According to a survey of rheumatologist by Childhood Arthritis and Rheumatology Research Alliance (CARRA), the changes in immune therapy that were made after the diagnosis of calcinosis were: 57% rheumatologists added IVIg, 48% added pulse steroids, 40% added methotrexate if the patient was already not on it, 25% survey responders added TNF-alpha inhibitors, 19% added rituximab, 11% added abatacept, and 12% used other immunosuppressants (anakinra, cyclophosphamide, lenalidomide, sirolimus). Of all the survey responders, 32% believed pulse steroids as a most successful option, 30% believed that IVIg was the most successful treatment option, and 29% believed IVIg was the second-best (after steroids) treatment option. The non-immunosuppressant treatment options by frequency of use included bisphosphonates (73%), calcium channel blockers (43%), topical formulation (14%), sodium thiosulfate (10%) and other agents such as warfarin, minocycline (4%) [50].

## Conclusions

The understanding of the etiology and pathogenesis of idiopathic immune myositis is fast evolving, and so is the classification of myositis subtypes. The diversity in genetics, major histocompatibility complex expressions, immunohistochemical, and specific and associated autoantibodies begs for subtype-specific treatment options for specific subtypes of IIM. The diversity in subtypes of IIM also explains the individual variability in response to specific therapies. The recent advances in biological therapies have made the notion of subtype-specific therapy option a possibility. Despite all these new advances in the knowledge and understanding of IIM and the novel biologics therapies, IVIg remains relevant, valid, unreplaceable treatment option in the management of certain subtype of IIM patients and certain specific clinical scenarios. It is an effective therapy in infection-associated myositis, IIM with dysphagia/dysphonia/IIM-associated ILD, refractory IIM and IIM with dystrophic calcinosis. Its utility in refractory IIM skin diseases has been long established. Apart from the efficacy, the tolerability and safety profile of IVIg remains unmatched to date.
